# Physico‐Chemical Characteristics, Sensory Properties and Microbial Quality of Date Fruit Added *Rasomalai* Prepared From Buffalo's Milk

**DOI:** 10.1002/fsn3.71145

**Published:** 2025-11-12

**Authors:** S. M. Rubayet Ferdous Rupom, Md. Sadman Tahmid, Gautam Kumar Deb, Md. Ashadul Alam, Md. Rakibul Hassan, Arifur Rahman, Anzuman Ara, Mohammad Shohel Rana Siddiki, Md. Harun‐ur‐Rashid, Tahir Mahmood Qureshi, Mohammad Ashiqul Islam

**Affiliations:** ^1^ Department of Dairy Science Bangladesh Agricultural University Mymensingh Bangladesh; ^2^ Bangladesh Livestock Research Institute Dhaka Bangladesh; ^3^ Department of Dairy Science Sylhet Agricultural University Sylhet Bangladesh; ^4^ Department of Food Sciences Cholistan University of Veterinary and Animal Sciences Bahawalpur Pakistan

## Abstract

*Rasomalai* is an indigenous popular dessert made from *chhana* (acid curd) balls, which are cooked in concentrated sugar syrup, and further soaked in sweetened condensed milk or *malai* (volume of milk reduced to about 50% of the original). Milk and dairy products do not contain dietary fiber. In the present study, incorporation of dietary fibers was carried out by the addition of 0%, 3%, 6%, and 9% of dates *(Phoenix dactylifera*) into *rasomalai* prepared from buffalo's milk. The main goal of the present study was to assess the physico‐chemical and sensory characteristics and microbiological quality of dates added buffalo milk *rasomalai*. The acidity of all the *rasomalai* types was ranging from 0.11% to 0.13%, and pH ranged from 6.08 to 6.66. We observed a linear increase in the total dietary fiber content of the *rasomalai* with the increase in the level of added dates, which ranged from 3.44% in the 3% dates to 13.38% in the 9% dates added *rasomalai*. The microbial quality was found statistically similar in all four groups of *rasomalai*. It was observed that the addition of date fruit intensified red and yellow hues in *rasomalai* while diminished its overall lightness. Notably, in the sensory evaluation, based on a 9‐point Hedonic Scale, the overall appearance score ranged from 6.33 to 8.0 in the prepared *rasomalai*; however, the highest score for both appearance (8.0) and taste/flavor (8.68) was recorded for the 3% dates added sample. These findings open doors for the development of healthier, more appealing *rasomalai* variants that cater to both the palate and nutritional well‐being.

## Graphical abstract

Different level of dates were added to the buffalo milk *rasomalai* to enhance its total dietary fiber content. The physicochemcial, microbial, sensorial, color and consumers likings were assessed in response of this dates addition. The addition of dates enrich the *rasomalai* with the total dietary fiber without affecting the chemical and microbial qualities and consumer accepted these types of *rasomalai*.

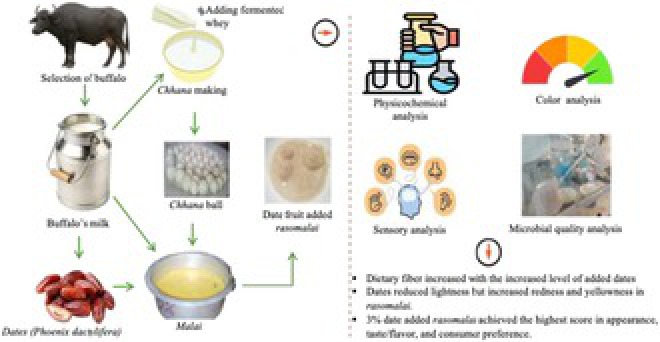

## 1 Introduction

Milk offers a well‐balanced array of lipids, proteins (including caseins and whey proteins), carbohydrates (primarily lactose), minerals like calcium and phosphate, enzymes—lysozyme, lactoperoxidase, xanthine oxidase, lipase, alkaline phosphatase, ribonuclease and protease, vitamins—vitamin A, vitamin B_12_ and vitamin E, and trace elements—potassium, magnesium, manganese, zinc, iron and copper (Jonas and Proctor 2018). Since time immemorial, milk has been processed into various products depending on regional preferences. Bangladesh, India, Pakistan and other neighboring countries adapted artisanal techniques for the conversion of highly perishable milk into dairy products having extended shelf‐life. The traditional dairy products of this region include fat‐rich products (clotted cream, *makhon*, butter, and ghee), concentrated products (khoa and khoa‐based sweetmeats, and rabri), heat‐acid coagulated products (*rosogolla*, *monda*, *rasomalai*, and *ponir*), fermented products (*doi/dahi/dadhi* and *lassi*), and frozen products (*kulfi*, *dudh malai* and *kulfa*) (Asif et al. 2021; Islam et al. 2003). Sweetmeat is a common term encompassing several different dairy products, mostly made of heat‐acid coagulated milk curd, *chhana*. Milk *chhana* is characterized by its high milk solids, especially milk fat, protein and mineral contents. It takes the spotlight as the key ingredient in a number of sweet delicacies found throughout Bangladesh (Islam et al. 2003), viz. *rosogolla*, *rasomalai*, *kalojam*, *lalmohon*, *chomchom*, *sandesh*, etc. The quality of *chhana* is influenced by factors like milk type and processing factors viz. heat treatment of milk, coagulants used, acidity/pH of the added coagulant, residence time in the milk‐coagulant mix, straining method and duration etc. (Jonkman and Das 1993). However, *chhana* from cow and buffalo milk possesses acceptable flavor, though the buffalo milk *chhana* making needs some modifications compared to the cow's one (Ahmed et al. 2018). In India, 40% of milk production is directed towards indigenous products, with approximately 4% allocated to *chhana* production (Mathur and Singh 2001). Similarly, about 4% of the total milk output in Bangladesh is used for *chhana* production which is eventually used for making sweetmeat (Islam and Basak 2013). Sometimes, sweetmeats are termed as sweet cheese, as sugar syrup is used for cooking and/or storage, or they contain high sugar in their formulation or both (Asif et al. 2021).

*Rasomalai* is a dessert‐type dairy product originated from the eastern part of the Indian subcontinent namely the Bengal region. It is prepared by cooking *chhana* balls in the sugar syrup (≈60°Brix) and eventually dipped into the *malai* (heat concentrated milk; volume reduced by at least 50%). According to Sharma et al. (2015), condensed milk can also be used instead of heat concentrated milk *malai*. It is one of the most preferred sweetmeats in Bangladesh, India and Pakistan (Qureshi et al. 2024). The preparation of *rasomalai* as a sweetmeat in Bangladesh primarily involves cow's milk, buffalo's milk, or a combination of both. However, Sayedatunnesha et al. (2008) advocated buffalo's milk for *rasomalai* production. Asif et al. (2021) and Qureshi et al. (2024) used buffalo's milk, whereas, Sharma et al. (2014) used both cow's and buffalo's milk in their study on *rasomalai*. The quality and quantity of *rasomalai* were better or improved if paneer/*ponir*/*chhana* balls were prepared from buffalo's milk rather than cow's milk.

The milk type and non‐dairy ingredients used are critical in manufacturing dairy products including *rasomalai* with regards to typical sensory attributes and nutritional quality. However, consumers consider the nutritional features of dairy products before making any buying decisions. Milk has no dietary fiber which has important beneficial health effects including gut and heart health (Drewnowski et al. 2021; Ibrahim et al. 2024). Dates are an important source of dietary fiber, readily metabolizable natural sugars, protein, vitamins, minerals, and other natural antioxidant complexes such as carotenoids and phenolics. Dates are also valued as an exceedingly healthful food with several different nutraceutical properties (Ibrahim et al. 2024). It has been reported that Muzafati dates contain 55.51% total sugar and a significant amount of vitamin C (1.40 mg/100 g) (Qureshi et al. 2025). Non‐dairy ingredients are also being added into ice cream and processed cheese by the dairy industry. Likewise, date fruit as a source of dietary fiber in the *rasomalai* needs to be analyzed concerning sensorial and physico‐chemical properties. Ambili et al. (2023) use herbs in the preparation of *rasomalai* and reported a significant variation between the control and herbal *rasomalai* with regard to the sensory parameters, i.e., flavor, body and texture and overall acceptability. In another study, Islam et al. (2015) suggested that 25% soy milk *chhana* can be mixed with 75% of the cow milk *chhana* during the preparation of *rasomalai*. However, we could not come across any study reporting instrumental color profile, descriptive sensorial analyses and consumers' liking hedonic scale results.

Moreover, the availability of scarce literature over *rasomalai* indicates that there are still ample opportunities to contribute to the field of *rasomalai* especially, prepared with buffalo's milk. From the scarce literature evidence, it was assumed that the addition of date fruit in *rasomalai* will influence its qualitative characteristics. Therefore, the objective of the present study was to assess the impact of adding different proportions of date fruit on the physico‐chemical and sensory properties, and the microbial quality of buffalo's milk *rasomalai*.

## 2 Materials and Methods

### 2.1 Experimental Site and Raw Materials

This experiment was conducted at the Laboratory of Dairy Chemistry and Technology (LDCT), and Laboratory of Dairy Microbiology and Biotechnology (LDMB), Department of Dairy Science, Bangladesh Agricultural University (BAU), Mymensingh. Fresh buffalo's milk was collected from BAU dairy farm and was analyzed immediately. Milk was kept in the refrigerator (5°C) for 5 ± 2 h and the fat‐rich top layer of milk was collected after every 20 min for *malai* preparation. The whole milk was fractionated at 1:2 ratio for *chhana* and *malai* preparation, respectively. Fat‐rich top layer milk was kept for *malai* making and the rest of the milk was used for *chhana* making. All types of milk quality were assessed by using the Laktan Ultrasonic Milk Analyzer (1–4 M 600 ULTRA, Russia) and the results are presented in Table 1.

**TABLE 1:** Chemical composition (mean ± SD) and somatic cell count (SCC, × 10^5^/mL) of buffalo's milk used for *rasomalai* preparation.

Buffalo milk	Density	Total solids %	Fat %	Solids‐not‐fat %	Lactose%	Protein%	SCC
Fresh milk	30.88 ± 4.11	16.49 ± 1.19	7.76 ± 0.82	8.99 ± 0.66	4.98 ± 0.42	3.3 ± 0.25	1.90 ± 0.03
Milk for *Malai*	27.13 ± 2.35	15.41 ± 1.88	9.39 ± 0.55	6.61 ± 0.51	4.89 ± 0.31	2.95 ± 0.28	—
Milk for *Chhana*	30.22 ± 0.93	16.13 ± 1.33	4.77 ± 0.66	7.07 ± 2.5	4.71 ± 0.28	3.72 ± 0.11	—

Dates ( *Phoenix dactylifera* ), sugar, cardamom, and wheat flour were purchased from the KR market, BAU, Mymensingh‐2202, Bangladesh. Fermented whey water (acidity ≈0.70% LA) was obtained from Boyra Bepari para sweetmeat shop, Boyra, Mymensingh, Bangladesh.

### 2.2 Preparation of *Rasomalai*

**FIGURE 1:**
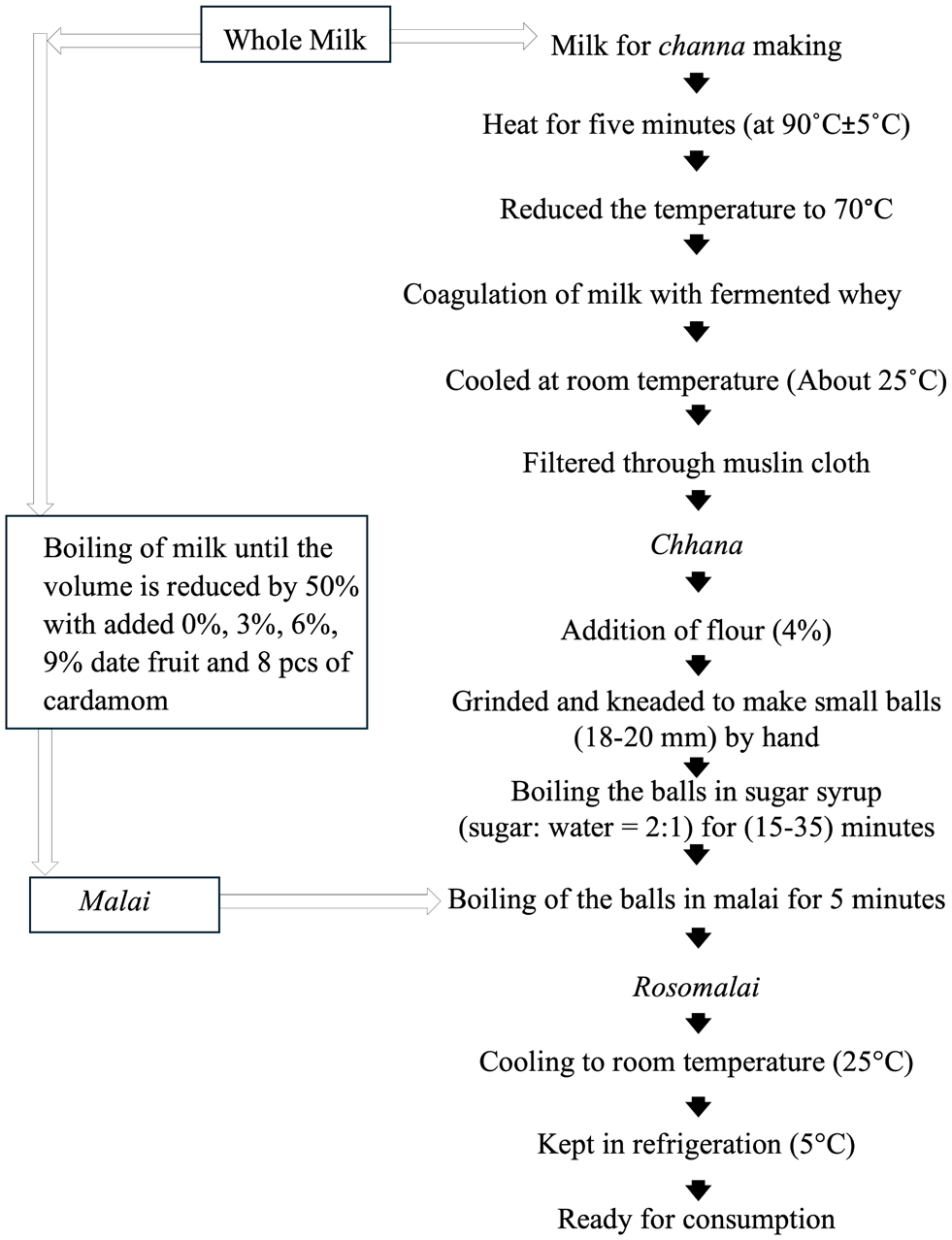
Schematic diagram for the manufacturing process of date fruit added *rasomalai* (modified from Asif et al. 2021).

The *rasomalai* was prepared following the methods described by Asif et al. (2021) with some modifications. The manufacturing flow diagram is illustrated in Figure 1.

#### 2.2.1 Preparation of *Chhana* Ball

Buffalo's milk (3.3 L) was heated until 90°C in a wide and heavy‐bottomed pan. Afterwards, it was kept at room temperature and when the temperature of the milk reduced to 70°C, fermented whey water was added until the milk curd and greenish whey were separated. The whole contents were then strained through a muslin cloth for complete drainage of whey water, and subsequently, the *chhana* (the filtered mass of heat‐acid curd of milk) was weighed and kneaded (with 4% added wheat flour, w/w) to obtain a smooth dough. The moisture content (%) of the prepared *chhana* was ≈50%. The dough was then eventually worked with the palms of both hands to shape it like a marble ball.

#### 2.2.2 Preparation of *Malai*

The milk allocated for the *malai* was portioned into four equal parts, each measuring 1660 mL. The milk was heated separately, and during boiling, blended dates were added at different levels: 0% date fruit = T_0_; 3% date fruit = T_1_; 6% date fruit = T_2_; and 9% date fruit = T_3_. The heating was continued with continuous stirring till the volume got reduced by 50%. Additionally, 6–8 crushed cardamom pods were added a few minutes before the end of heating. The prepared *malai* was kept ready for the dipping of the *chhana* balls.

#### 2.2.3 Cooking of *Chhana* Ball and Dipping in *Malai*

*Chhana* balls were cooked in the sugar syrup. For this purpose, 50% sugar syrup (w/w) was used and a small amount of milk was added to the sugar syrup during its preparatory heating to make it scum‐free and clean. The *chhana* balls were cooked in the sugar syrup for 15–35 min based on the heating intensity. After cooking the *chhana* balls, they were dipped into the boiling *malai*. This step imparts richness to the *rasomalai*. Three batches of *rasomalai* of each treatment group were prepared. The *rasomalai* was allowed to cool to room temperature and kept overnight in a refrigerator (5°C) for sensorial evaluation or at −20°C for further analyses.

### 2.3 Physico‐Chemical Properties, Chemical Composition and Microbial Quality

#### 2.3.1 Acidity Test and Color Analysis

The acidity of *rasomalai* was assayed by titration against 0.1 N NaOH using phenolphthalein as an indicator (AOAC 2016, **981.12**). For color profiling, Konica Minolta Colorimeter (Minolta Laboratories, Osaka, Japan) was used. The profiling includes luminosity (L*) axis i.e., lightness to darkness, scale from 0 (black) to 100 (white); and two axes of chromaticity viz. a*, which signifies green (−128 to 0) to red (0 to +127) and b* represents blue (−128 to 0) to yellow (0 to +127). For calculating the color difference between the samples, the following formula was used (Barukčić et al. 2022) –

$$\mathrm{\Delta E}={\left[{\left(L_{2}–L_{1}\right)}^{2}+{\left(a_{2}–a_{1}\right)}^{2}+{\left(b_{2}–b_{1}\right)}^{2}\right]}^{0.5}$$

where ΔE = Change in color as measured.

L = Lightness (L*); a = Redness (a*); b = Yellowness (b*).

For interpreting the color differences, the scale reported by Wild (2025) with remarks was used, which was –0: no color difference; 1–2: small color differences; 2–4: noticeable color differences; 4–10: significant color differences; 10+: very large color differences.

#### 2.3.2 Chemical Composition of *Rasomalai*

For total solids content, the *rasomalai* samples were placed in a hot air oven (WTB Binder Labortechnik GmbH, D‐78532 Tuttlingen, Germany) for 24 h at 105°C (AOAC 2016, **925.10**). The dried samples were ignited (at 600°C) for 5 h in a muffle furnace (VULCAN A550 Furnace, USA) in order to estimate the ash content of the *rasomalai* (AOAC 2016, **923.03**). The Babcock fat test method was used for assaying the fat content of the *rasomalai*. The crude protein of *rasomalai* was determined by the Kjeldahl method (AOAC 2016, **984.13**). The total dietary fiber (TDF) was measured according to AOAC (2016, **962.09**) at the Department of Food Processing and Preservation (FPP), Hajee Mohammad Danesh Science and Technology University, Dinajpur‐5200, Bangladesh. In brief, the sample was defatted using hexane followed by drying in a vacuum desiccator. Then 500 mg of dried samples were added to 25 mL deionized water in a Teflon tube. The tube was closed and heated at 121°C for 1.25 h in a hot air oven. Then it was allowed to cool a bit (≈65°C–70°C), followed by the addition of 2 mL of 0.15 mL amyloglucosidase, 1 mL acetate buffer, and 0.85 mL water mixture. After vigorous shaking, it was heated for another 2 h at 55°C with occasional agitation. The contents were transferred to a beaker and 110 mL of 95% ethanol was added to it and allowed to precipitate for 60 min at room temperature. Dilute alcohol‐insoluble material was collected on fritted crucibles using gentle suction. The residues were washed twice with 20 mL 78% ethanol, twice with 10 mL 95% ethanol, and once with 10 mL acetone. The crucibles with residues were dried at 105°C overnight. The ash of the residues was obtained by heating at 525°C for 5 h. The protein of the residues was assayed by using a modified ammonia determination procedure. Blank samples were also run in the same way.

#### 2.3.3 Standard Plate Count (SPC)

For SPC, serial dilutions of *rasomalai* samples were made and plated onto SPC agar plates following the protocol of Khademi et al. (2022). The plates were incubated at 37°C for 48 h. Then the counts were made.

### 2.4 Sensory Evaluation

A discerning sensory panel, comprising 15 individuals (6 females and 9 males; aged between 20 and 53 years) possessing substantial expertise in the assessment of dairy products evaluated the *rasomalai* concerning its descriptive sensorial properties. The evaluation was approved by the panel of the Department of Dairy Science, Bangladesh Agricultural University. The selection of sensory panelists was based on their ability to discern the four fundamental tastes (sweet, sour, salty and bitter). During panel training, a specific reference frame for assessor training was established by attaching three scale points (representing low, medium, and high intensity) to the reference material, as outlined in Table 2. The reference definition was modified from Uzun et al. (2018) as presented in Table 3. The samples were coded and randomly distributed among the panelists.

**TABLE 2:** Panelists' descriptive scale (Uzun et al. 2018).

Scale	Score
Low	0–35 (points)
Moderate	36–70 (points)
High	71–100 (points)

*Note:* 0, absent; 100, very strong.

**TABLE 3:** Outlines of the descriptive attributes used for *rasomalai* evaluation.

Descriptor	Definition
*Appearance*	
*Color*	Overall intensity of the *Color* (*Chhana* ball‐ off white to creamy white: *Malai*–shades of white *Color* (seashell‐antique white‐linen) with slightly yellowish and/or brownish and/or caramelized tint)
Brightness	Overall intensity of light reflected from the exterior surface
Smoothness	Overall uniformity of the exterior surface
Internal Appearance	Overall uniformity of internal surface upon spoon cutting of *chhana* ball
Phase Separation	Overall homogenous ratio of *chhana* ball and *malai* and any watery thickness in any part of the *malai*.
*Flavor/odor*	
Overall odor	Overall odor intensity
Overall flavor	Overall flavor intensity
Milk	Flavor/odor arising from milk at ambient temperature
Whey	Flavor/odor related to whey
Mouthfeel	Overall intensity of texture‐ stickiness‐ density‐ moisture‐ and cooling/warming sensations
Caramelized flavor	Overall intensity of nutty flavor‐ crunchy texture and aroma of toasted sugar and caramel
Cooked	Overall intensity of sweet, sulfurous, and caramelized notes
Date	Overall earthiness‐ fruity undertone‐ contributing to a slightly thicker‐ pleasantly rich sweetness and chewiness.
*Taste*	
Sour	Fundamental taste associated with acid whey (Lactic Acid Whey)
Sweet	Fundamental taste related to sucrose
Bitter	Fundamental taste associated with poor quality or expired milk; overcooked *chhana* ball and/or *malai*
Salty	Fundamental taste associated with table salt (sodium chloride)
*Texture*	
Tenderness	The minimum amount of force required to chew *rasomalai* samples: higher tenderness results in lower force requirement
Juiciness	Moisture released during mastication (high: liquids are abundantly produced during mastication; low: saliva is absorbed by the product)
Cohesiveness	The degree to which a *rasomalai* sample holds together while being chewed
Chewiness	Easiness in masticating the sample to a state prior to swallowing
Consistency	Overall thickness, firmness or viscosity of *malai* and *chhana* ball
Grainy Texture	Presence of fine‐ grainy particles

Furthermore, thirty (30) untrained consumers aged between 22 and 55 years were also selected to assess the consumer preferences for date fruit added *rasomalai*. These participants were asked to express their overall liking for the samples and to rate four distinct aspects: overall liking, texture, appearance, and taste/flavor. A 9‐point hedonic scale was provided to the participants for this purpose.

### 2.5 Yield and Cost of *Rasomalai*

In order to calculate the cost of producing 1 kg of *rasomalai*, the following cost items in Table 4 were considered.

**TABLE 4:** Cost for the production of 1 kg date added *rasomalai*.

Ingredients	Unit cost (USD)	Date			
		0%	3%	6%	9%
Milk (kg)	0.58	1.08	1.05	1.01	0.99
Sugar (kg)	1.07	0.53	0.53	0.53	0.53
Date (kg)	4.93	0	0.18	0.35	0.51
Cardamom (kg)	20.54	0.062	0.062	0.062	0.062
Labor	4.11	0.41	0.41	0.41	0.41
Depreciation (3%)	0.24	0.049	0.049	0.049	0.049
Fuel	0.99	0.25	0.25	0.25	0.25

The yield (%), cost of production (USD/Kg), profit margin (USD/Kg) and cost–benefit ratio of *rasomalai* was calculated by using the following formulae, respectively

$$1.\;\text{Percent yield}=\frac{\text{Total yield of}\;\text{rasomalai}}{\text{milk used in}\;\mathrm{kg}+\text{Sugar used in}\;\mathrm{kg}+\text{date used in}\;\mathrm{kg}+\text{Cardamom used in}\;\mathrm{kg}}\times 100$$

$$2.\;\text{Cost of production}\;\mathrm{per}\;\mathrm{kg}=\frac{\text{Cost of production}\;\left(\mathrm{USD}\right)}{\text{Yield of}\;\text{rasomalai}\;\left(\mathrm{kg}\right)}$$

$$\begin{array}{c} 3.\;\text{Profit}\;\left(\mathrm{USD}/\mathrm{kg}\right)=\text{Selling price of}\;1\mathrm{kg}\;\text{rasomalai}\\ \;-\text{Production cost of}\;1\mathrm{kg}\;\text{rasomalai} \end{array}$$

$$4.\text{Cost‐Benefit}\;Ratio\;=\frac{Profit\;(USD)}{\text{Cost of production}\;\left(\mathrm{USD}\right)}$$

It was surveyed that the market price of *rasomalai* in Bangladesh is about 4.11USD per kg.

### 2.6 Statistical Analysis

The data collected in the study concerning physico‐chemical, microbiological and sensory analyses were analyzed using one‐way analysis of variance by employing Minitab‐17 statistical software. In case of a significant difference, mean separation was done by Tukey's test.

## 3 Results

### 3.1 Physico‐Chemical Properties and Microbial Quality

The results concerning acidity, and chemical composition are presented in Table 5. The inclusion of date tends to impart a bit higher acidity in the *rasomalai*; however, the range is very low (0.11%–0.14% LA) and differed non‐significantly (*p* > 0.05). The total solids content of the *rasomalai* with different levels of date addition showed significant (*p* < 0.05) variations. *Rasomalai* with 9% added date had 7% more total solids than the *rasomalai* without date. Even though the total solids of *rasomalai* having date addition of 3% were 6% higher than the *rasomalai* samples having no date fruit addition, those samples were statistically similar to the *rasomalai* having 9% dates. Addition of 6% date fruit gave 47% total solids in the *rasomalai* which was 5% higher (*p* < 0.05) than the *rasomalai* without date fruit. In contrast to the total solids, fat content showed an opposite trend in response to date addition. Fat content (10%) of the *rasomalai* with 3% and 6% date fruit addition was only 1% lower than the *rasomalai* having 0% date fruit addition. Interestingly, *rasomalai* having 9% date added only had 7% fat but all the samples showed non‐significant variations (*p* > 0.05). All the samples showed protein contents in the range of 13%–14% but the protein contents did not vary significantly (*p* > 0.05) among the treatments. The higher levels (6% and 9%) of the date addition into *rasomalai* caused only a 1% reduction in the protein contents compared to the lower two levels (0% and 3%) of date addition to the *rasomalai*. The crude protein contents of 0% and 3% date‐added *rasomalai* were the highest (14%) and, 6% and 9% date‐added *rasomalai* CP contents were 13% (*p* > 0.05). The total dietary fiber content of the *rasomalai* showed a significant increase with the increased level of date addition (*p* < 0.05). The highest dietary fiber (10%) was recorded in the 9% date added *rasomalai* which was 1.5 and 3 folds greater compared to 6% and 3% date added *rasomalai*, respectively. Moreover, the highest total dietary fibers (TDF) were observed in 9% date *rasomalai* (10.33%) while the TDF were absent in *rasomalai* samples having 0% dates. The highest carbohydrate contents were also found in the 9% date fruit added *rasomalai* (25%) which was significantly higher (*p* < 0.05) than the other *rasomalai* samples. Concerning TDF and carbohydrate, though the 3% and 6% date added *rasomalai showed similar carbohydrate content but the TDF is almost double in 6% than that of the 3% date added rasomalai*. The ash contents of date fruit added *rasomalai* were not significantly (*p* > 0.05) varied and ranged from 1.33% to 1.50%.

**TABLE 5:** Comparison of physico‐chemical and microbial attributes (mean ± SD) among the date‐added buffalo milk *rasomalai*.

Physico‐chemical parameters	0% date	3% date	6% date	9% date	*p*
Acidity%	0.11 ± 0.01	0.13 ± 0.01	0.13 ± 0.002	0.14 ± 0.02	0.130
Total Solids%	42.13^c^ ± 0.30	48.29^a^ ± 0.41	47.07^b^ ± 0.73	49.21^a^ ± 0.25	0.000
Fat%	11.0 ± 01.80	10.17 ± 02.02	10.0 ± 3.12	7.42 ± 1.13	0.271
CP%	14.51 ± 01.49	14.22 ± 01.11	13.30 ± 01.44	13.38 ± 1.06	0.601
TDF%	—	3.44^c^ ± 0.48	6.89^b^ ± 0.96	10.33^a^ ± 01.44	0.001
CHO and others	14.32^c^ ± 0.60	20.87^b^ ± 1.25	20.63^b^ ± 1.82	25.39^a^ ± 1.57	0.000
Ash%	1.33 ± 0.03	1.49 ± 0.12	1.43 ± 0.07	1.47 ± 0.07	0.150
SPC (CFU/g)	30.0 ± 6.52	39.67 ± 8.62	36.33 ± 9.29	40.33 ± 13.05	0.728

*Note:* ^a,b,c^Means with different superscript (s) in a row are significantly different (*p* < 0.05).

Abbreviations: CHO, carbohydrates; CP, crude protein; SPC, standard plate count; TDF, total dietary fiber.

The standard plate counts (SPC) of date‐added *rasomalai* at different concentrations (0%, 3%, 6%, and 9%) are presented in Table 5. The SPC of control *rasomalai* (0%) was 30.00 ± 6.52 CFU/g, whereas, *rasomalai* having 9% date fruit had an increased number of SPC, i.e., 40.33 ± 13.05 CFU/g. However, these increments in SPC for all the treatments of *rasomalai* did not vary significantly (*p* > 0.05). It is not necessary to observe significant variations between treatments because date fruit do not add significant microorganisms in the *rasomalai* mixture.

**TABLE 6:** Color parameters of date *rasomalai* and control group.

Color parameter	0%	3%	6%	9%	*p*
L	94.69^a^ ± 1.58	81.03^b^ ± 3.97	80.64^b^ ± 1.94	80.36^b^ ± 0.77	0.000
a*	−3.46^b^ ± 0.42	1.83^a^ ± 1.49	4.41^a^ ± 1.29	4.64^a^ ± 1.13	0.000
b*	12.69^b^ ± 0.49	13.93^b^ ± 5.79	20.75^a^ ± 1.08	21.28^a^ ± 1.21	0.015

*Note:* L*, luminosity axis i.e., lightness to darkness (0 (black) to 100 (white)); a*, green (−128 to 0) to red (0 to +127); b*, blue (−128 to 0) to yellow (0 to +127); ^a,b^Means with different superscript(s) in a row are significantly different (*p* < 0.05).

### 3.2 Color Tests

Table 6 shows the results concerning color parameters, i.e., lightness, redness, and yellowness. The control samples of *rasomalai* (0% dates) showed 95 unit of brightness which was 14 unit higher than the date‐added samples. The lightness values were significantly (*p* < 0.05) decreased by the subsequent increase in the quantities of date fruits in *rasomalai*. The redness and yellowness of *rasomalai* were subsequently increased (*p* < 0.05) by the increase in the quantities of dates. The maximum redness (4.64) and yellowness (21.28) were shown by *rasomalai* having 9% dates. In contrast, the lowest values of redness and yellowness were found in control *rasomalai* (0% dates). This variation is much more reflected in Table 7 where the color difference from the 0% products increased with the increased level of added dates. Among the other added levels, the differences were also found more when the differences between the added levels were more except 6% vs 9%.

**TABLE 7:** Color differences among the *rasomalai* samples made from different levels of added dates.

	0% vs. 3%	0% vs. 6%	0% vs. 9%	3% vs. 6%	3% vs. 9%	6% vs. 9%
ΔE	14.70	18.01	18.57	7.30	7.90	0.64

*Note:* ΔE = [(L2–L1)^2^ + (a2–a1)^2^ + (b2–b1)^2^] 0.5 (Barukčić et al. 2022); 0, no color difference; 1–2, small color differences; 2–4, noticeable color differences; 4–10, significant color differences; 10+, very large color differences (Wild 2025).

### 3.3 Sensory Properties

The sensory characteristics of *rasomalai* samples enriched with varying levels of date were evaluated and compared using descriptive analysis (Table 8). The descriptive parameters of sensory properties were color, brightness, smoothness, internal appearance, phase separation, overall odor, overall flavor, milk flavor, whey flavor, mouthfeel, caramelized flavor, cooked flavor, date flavor, sourness, sweetness, bitterness, saltiness, tenderness, juiciness, cohesiveness, chewiness, consistency and grainy texture. Among the evaluated sensory attributes, most of them showed statistically non‐significant differences (*p* > 0.05) across the treatments. However, a notable exception was observed in date flavor, which differed significantly (*p* = 0.000) among treatments. The control sample (0%) scored 0 for date flavor, while the 3%, 6%, and 9% date‐fortified samples scored progressively higher. This result indicated the effective incorporation and increasing perception of date flavor with higher concentrations at 9% date‐added *rasomalai* (73.33 ± 16.07). This trend is expected and confirms the contribution of date addition to the flavor profile of *rasomalai*.

**TABLE 8:** Comparison of descriptive sensory properties of date‐added *rasomalai*.

Parameters	0%	3%	6%	9%	*p*
*Color*	70.67 ± 5.13	70.0 ± 10.0	61.67 ± 12.58	56.67 ± 15.28	0.418
Brightness	71.67 ± 7.64	70.0 ± 05.0	51.70 ± 25.70	51.70 ± 25.70	0.421
Smoothness	69.0 ± 13.53	61.67 ± 5.77	66.67 ± 15.28	70.0 ± 13.23	0.849
Internal Appearance	68.33 ± 2.89	60.67 ± 7.51	56.0 ± 24.20	55.0 ± 22.90	0.772
Phase separation	73.33 ± 2.89	63.33 ± 11.55	65.0 ± 15.0	64.0 ± 13.0	0.622
Overall odor	83.33 ± 11.55	75.0 ± 8.66	71.67 ± 7.64	74.0 ± 10.15	0.509
Overall flavor	81.67 ± 10.41	70.0 ± 10.0	74.33 ± 13.65	74.33 ± 13.65	0.703
Milk flavor	85.67 ± 13.65	78.33 ± 14.43	41.67 ± 36.85	42.70 ± 37.20	0.208
Whey flavor	5.83 ± 3.69	14.0 ± 14.42	3.33 ± 5.77	06.0 ± 05.29	0.477
Mouthfeel	73.33 ± 14.43	66.67 ± 7.64	76.67 ± 07.64	76.67 ± 07.64	0.580
Caramelized flavor	25.67 ± 5.13	41.70 ± 17.60	40.33 ± 9.61	27.0 ± 15.72	0.343
Cooked	48.30 ± 28.90	45.0 ± 26.0	45.0 ± 32.80	35.70 ± 38.40	0.965
Date flavor	0.0^b^	63.33^a^ ± 7.64	70^a^.0 ± 10.0	73.33^a^ ± 16.07	0.000
Sour	3.0 ± 5.20	3.33 ± 5.77	3.33 ± 2.89	6.67 ± 2.31	0.702
Sweet	75.0 ± 21.80	71.70 ± 20.20	76.67 ± 7.64	76.67 ± 7.64	0.976
Bitter	5.67 ± 4.16	4.67 ± 4.73	4.0 ± 1.73	6.67 ± 2.31	0.797
Salty	15.0 ± 8.66	21.67 ± 7.64	9.67 ± 5.03	8.33 ± 6.11	0.160
Tenderness	70.0 ± 05.0	68.33 ± 10.41	71.30 ± 17.90	70.67 ± 16.77	0.994
Juiciness	76.67 ± 10.41	63.33 ± 15.28	81.67 ± 2.89	84.33 ± 9.29	0.144
Cohesiveness	55.0 ± 13.23	60.0 ± 18.0	55.7 ± 34.0	54.0 ± 31.50	0.992
Chewiness	68.33 ± 10.41	85.0 ± 10.0	70.0 ± 05.0	70.0 ± 10.0	0.166
Consistency	75.0 ± 05.0	80.67 ± 9.02	73.33 ± 1.53	72.67 ± 2.52	0.318
Grainy Texture	68.33 ± 10.41	58.33 ± 10.41	53.0 ± 09.64	55.67 ± 12.10	0.376

*Note:* ^a,b^Means with different superscripts in a row are significantly different (*p* < 0.05).

Moreover, Table 9 presented the results of the consumers liking of prepared *rasomalai* samples. It was observed that 3% date fruit added *rasomalai* had significantly (*p* < 0.05) the highest sensory scores on taste/flavor as compared to all other levels; however, overall appearance, texture, and overall liking scores were found similar (*p* > 0.05).

**TABLE 9:** Comparison of consumers' liking of date‐added buffalo milk *rasomalai*, using hedonic scale (9 points).

Sensory	0%	3%	6%	9%	*p*
Overall appearance	6.67 ± 0.577	8.0 ± 1.0	7.0 ± 0.0	6.33 ± 0.58	0.060
Taste/flavor	6.68^b^ ± 0.58	8.68^a^ ± 0.58	8^a,b^.0 ± 1.0	6.33^b^ ± 0.58	0.011
Texture	7.0 ± 1.0	8.33 ± 0.58	7.33 ± 0.58	6.68 ± 0.58	0.089
Overall liking	7.33 ± 0.58	7.68 ± 1.53	7.33 ± 0.58	6.67 ± 1.53	0.760

*Note:* ^a,b^Means with different superscripts in a row are significantly (*p* < 0.05) different.

### 3.4 Yield and Costing

Table 10 summarizes the yield, cost of production, cost–benefit ratio, and profit margin of buffalo's milk *rasomalai*. The cost of production (USD/kg) increased progressively (*p* < 0.05) with increasing levels of date addition, from 2.39 ± 0.044 USD in the control to 2.85 ± 0.21 USD in the 9% date added *rasomalai* sample. It can be seen that the *p*‐values (*p* = 0.012) denote statistically significant differences in cost of production and profit margin.

**TABLE 10:** Yield and cost of date‐added buffalo milk *rasomalai*.

Parameters	0%	3%	6%	9%	SE of difference	*p*
Percent yield	44.35 ± 1.26	44.83 ± 1.37	45.69 ± 1.62	45.90 ± 2.20	1.35	0.643
Cost of production (USD/kg)	2.39^b^ ± 0.044	2.53^a,b^ ± 0.053	2.66^a,b^ ± 0.12	2.85^a^ ± 0.21	0.10	0.012
Cost benefit ratio	0.014	0.013	0.013	0.012	—	—
Profit margin (USD)	1.72^a^ ± 0.044	1.58^a,b^ ± 0.053	1.45^a,b^ ± 0.12	1.26^b^ ± 0.21	0.10	0.012

*Note:* ^a,b^Means with different superscripts in a row are significantly different (*p* < 0.05).

## 4 Discussion

### 4.1 Chemical Composition and Microbial Quality of Date Fruit Added Buffalo Milk *Rasomalai*

The results showed that the acidity was increased as the quantities of date fruit were increased in *rasomalai* whilst fat (%) was reduced by the subsequent increase in dates in *rasomalai*. Al‐Farsi and Lee (2008) stated that the fat percentage of dates ranged from 0.2% to 0.5%. So, the present findings suggest that the reduction in fat percentage in *rasomalai* with increased date addition can be attributed to the dilution of milk fat by the low‐fat, high‐fiber date pulp. This indicates that the addition of dates lowers the fat percentage of *rasomalai*. Sharma et al. (2015) observed that the acidity levels in *chhana* patties ranged from 0.30% to 0.47%, while the fat contents of *rasomalai* ranged from 9.20% to 10.97%. Present findings indicated that fat contents in *rasomalai* were slightly higher and acidity was slightly lower than in the study of Sharma et al. (2015). This difference can be attributed to the use of fresh *rasomalai* samples in the acidity assessment, which resulted in slightly lower acidity values in the current study. The fresh samples had a low number of microorganisms which did not cause any production of acids in the mixture of *rasomalai*. The acidity of *rasomalai* gradually increased during storage. Initially, all freshly prepared *rasomalai* samples exhibited lower acidity levels. However, the acidity was increased during storage due to the accumulation of acids produced by the activity of contaminating microorganisms. The addition of dates did not have any effect on the product's CP content. Buffalo's milk contains higher quantities of fat and solids‐not‐fat (SNF) as compared to cow's milk (Qureshi et al. 2025). They analyzed the physico‐chemical characteristics of date varieties viz. muzafati, popo, aseel and ajwa. According to them, date fruits contain 0.4 ‐ 1% fat and 0.8 ‐ 2.0% protein. By adding 3%, 6% and 9% dates to the *rasomalai*, fat or protein contents were reduced as the original mixture of *rasomalai* had higher fat contents as compared to date fruits. Moreover, the current study results were in accordance with the observations of Qureshi et al. (2024) who reported that higher fat and protein contents improved the quality of *rasomalai* and these contents predominantly depend on the raw milk (Tyagi et al. 2017). The total solids content decreased with the increase in dates addition in *rasomalai*. The addition of dates to *rasomalai* significantly influenced its compositional properties, particularly the total solids and ash content. Sharma et al. (2015) reported that the total solids and ash contents in *rasomalai* were in the range of 38.54%–40.30% and 1.26%–1.31% respectively, which were higher than observed in the current findings. Total solids increased with higher levels of date incorporation, which can be attributed to the rich carbohydrate, fiber, and mineral content of date fruits. These components directly contribute to the dry matter of the final product. A similar trend was observed for ash content, which also increased with date addition. This is due to the mineral richness of dates, especially in potassium, calcium, magnesium, and phosphorus. These findings are consistent with the report of Al‐Farsi and Lee (2008), who explained the high levels of dietary fiber and essential minerals in dates, supporting their role in enhancing the nutritional density of dairy‐based products.

The addition of dates influenced the microbial load in buffalo's milk *rasomalai*. Even though, variations in the SPC were present among different treatments of *rasomalai*, those variations were not statistically significant. Prodhan et al. (2017) reported that the standard plate count (SPC) in market *rasomalai* samples exceeded the permissible limit of 500 CFU/g, rendering them microbiologically unsuitable for human consumption. However, the current study's findings regarding SPC counts fall within acceptable levels for human consumption.

### 4.2 Sensorial Features

The color of dairy products provides consumers with insights into their sensory properties. The sensory attributes were evaluated to assess and quantify the product quality. The sensory properties of dairy products are primarily determined by the fat content of the milk, unless additional sensory enrichment is added from outside (Zielińska et al. 2020; Frøst et al. 2001). The inclusion of date pastes in buffalo's milk *rasomalai* influenced the overall appearance, taste, and flavor but did not have any effect on the texture and overall preference. Qureshi et al. (2025) observed significant variations in sensory properties of Pakistani dahi among different treatments (varying amounts of dates). The date fruit added *rasomalai* significantly varied only in taste/flavor, and 0%, 3%, 6%, 9% dates scored 6.68 ± 0.58, 8.68 ± 0.58, 8.0 ± 1.0 and 6.33 ± 0.58, respectively. In the present study, the highest sensory scores were observed in the *rasomalai* with 3% date fruit addition. The overall appearance scores were aligned with previous studies on *rosogolla* by Mohamed and Attala (2017) and Haque et al. (2003). Taste scores obtained in this experiment were comparable to those reported by Tarafdar et al. (2002) and Haque et al. (2003) for *rosogolla* made from cow's milk. Additionally, variations in texture scores among the samples were statistically non‐significant (*p* > 0.05) among treatments. The researchers Joshi et al. (1991) and Katiyar et al. (2008) noted that *chhana* prepared from buffalo's milk had a firmer body and coarser texture compared to *chhana* from cow's and goat milk. A similar trend was also observed in buffalo's milk *rasomalai*. The firm texture of *rasomalai chhana* balls prepared from buffalo's milk might be due to higher contents of proteins. The *chhana* balls prepared from cow's milk would have a less firm texture due to lower contents of protein as compared to buffalo's milk. Besides, the color of date fruit added *rasomalai* increased the redness and yellowness whereas a negative consequence on lightness was found in *rasomalai*. The color profile analysis of *rasomalai* or *chhana*‐based products is rare, but color analysis of Rennet curd‐based products can be found.

### 4.3 Yield and Costing

Cost is a crucial factor in every production process, influencing the producer's ability to make informed decisions. In comparison to previous research by Sayedatunnesha et al. (2008), the current study demonstrated higher yields, indicating that out of 2.5 kg of buffalo's milk, 1.3 kg of date fruit added buffalo's milk *rasomalai* could be obtained. Furthermore, the recent experiment findings revealed a higher profit margin in dates *rasomalai*. Although the addition of dates increased production costs due to their expensive nature, the higher yield compared to the control sample suggests that date fruit added *rasomalai* could be cost‐effective.

## 5 Conclusion

In this experiment, an endeavor was made to formulate one of the traditional dairy sweetmeats, *rasomalai* by incorporating dates into buffalo's milk. The results indicate that the addition of dates significantly enhanced the nutritional value of *rasomalai*. The addition of 9% date fruits into the *rasomalai* sample was found to be the best as this resulted in the highest total solids, TDF, CHO%, overall flavor, juiciness, and smoothness while maintaining acceptable values for other parameters but consumer acceptability decreased. In Contrast, *rasomalai*, having 3% date fruit, was the best in terms of taste/flavor properties, as well as the highest consumer acceptability based on the hedonic scale scores. The incorporation of dietary fiber in dairy products opens new avenues for health‐conscious consumers and proves economically advantageous, given the higher yield of *rasomalai* obtained from buffalo's milk. Therefore, the incorporation of fiber‐enriched buffalo's milk *rasomalai* could be a beneficial prospect.

## Author Contributions

**S. M. Rubayet Ferdous Rupom:** data curation (lead), formal analysis (lead), methodology (lead), validation (equal), visualization (equal), writing – original draft (equal), writing – review and editing (equal). **Md. Sadman Tahmid:** data curation (equal), formal analysis (equal), methodology (equal), validation (equal), visualization (equal), writing – original draft (equal), writing – review and editing (equal). **Gautam Kumar Deb:** conceptualization (supporting), funding acquisition (supporting), project administration (supporting), resources (supporting), writing – review and editing (supporting). **Md. Ashadul Alam:** data curation (supporting), funding acquisition (supporting), project administration (supporting), resources (supporting), validation (supporting), visualization (supporting), writing – review and editing (supporting). **Md. Rakibul Hassan:** data curation (supporting), supervision (supporting), validation (supporting), visualization (supporting), writing – review and editing (supporting). **Arifur Rahman:** data curation (supporting), formal analysis (supporting), investigation (supporting), methodology (supporting), software (supporting), visualization (supporting), writing – review and editing (supporting). **Anzuman Ara:** funding acquisition (supporting), project administration (supporting), resources (supporting), supervision (supporting), validation (supporting), writing – review and editing (supporting). **Mohammad Shohel Rana Siddiki:** formal analysis (supporting), funding acquisition (supporting), resources (supporting), writing – review and editing (supporting). **Md. Harun‐ur‐Rashid:** funding acquisition (supporting), project administration (supporting), resources (supporting), supervision (supporting), validation (supporting). **Mohammad Ashiqul Islam:** conceptualization (lead), funding acquisition (lead), project administration (lead), resources (lead), supervision (lead), validation (lead), writing – original draft (supporting), writing – review and editing (supporting).

## Conflicts of Interest

The authors declare no conflicts of interest.

## Data Availability

Data will be available at reasonable request.

## References

[fsn371145-bib-0001] Ahmed, S. , D. R. Menghwar , U. Qureshi , T. Ahmed , and S. Jakhrani . 2018. “Qualitative Studies on *Chhana* Prepared From Cow and Buffalo Milk.” Turkish Journal of Agriculture‐Food Science and Technology 6: 936–939. 10.24925/turjaf.v6i7.936-939.1925.

[fsn371145-bib-0002] Al‐Farsi, M. A. , and C. Y. Lee . 2008. “Nutritional and Functional Properties of Dates: A Review.” Critical Reviews in Food Science and Nutrition 48: 877–887. 10.1080/10408390701724264. 18949591

[fsn371145-bib-0003] Ambili, M. V. , D. Singh , S. N. Rajakumar , et al. 2023. “Shelf Life and Storage Studies on the Sensory Attributes of Dietetic Herbal.” Environment and Ecology 41: 321–325.

[fsn371145-bib-0004] Asif, A. H. M. , G. K. Deb , M. R. Habib , et al. 2021. “Variations in Fatty Acid and Amino Acid Profiles of Doi and *Rasomalai* Made From Buffalo Milk.” Journal of Advanced Veterinary and Animal Research 8: 511–520. 10.5455/javar.2021.h541. 34722751 PMC8520153

[fsn371145-bib-0005] Barukčić, I. , K. Filipan , K. Lisak Jakopović , R. Božanić , M. Blažić , and M. Repajić . 2022. “The Potential of Olive Leaf Extract as a Functional Ingredient in Yoghurt Production: The Effects on Fermentation, Rheology, Sensory, and Antioxidant Properties of Cow Milk Yoghurt.” Food 11: 701. 10.3390/foods11050701. PMC8909810 35267334

[fsn371145-bib-0006] Drewnowski, A. , C. J. Henry , and J. T. Dwyer . 2021. “Proposed Nutrient Standards for Plant‐Based Beverages Intended as Milk Alternatives.” Frontiers in Nutrition 8: 761442. 10.3389/fnut.2021.761442. 34746213 PMC8564006

[fsn371145-bib-0007] Frøst, M. B. , G. Dijksterhuis , and M. Martens . 2001. “Sensory Perception of Fat in Milk.” Food Quality and Preference 12: 327–336. 10.1016/S0950-3293(01)00018-0.

[fsn371145-bib-0008] Haque, A. , M. J. Alam , M. Hasanuzzaman , M. N. Islam , and M. A. Azad . 2003. “Comparison of Rosogolla Made From Fresh Cow Milk, Fresh Buffalo Milk and Mixture of Cow and Buffalo Milk.” Pakistan Journal of Nutrition 2: 296–299.

[fsn371145-bib-0009] Ibrahim, A. S. , R. Sukor , F. Anwar , S. Murugesu , J. Selamat , and R. Siva . 2024. “Nutritional, Nutraceutical Attributes, Microbiological and Chemical Safety of Different Varieties of Dates—A Review.” Future Foods 10: 100421. 10.1016/j.fufo.2024.100421.

[fsn371145-bib-0010] Islam, M. N. , F. Parvin , M. S. Hossain , A. Wadud , M. S. R. Siddiki , and M. S. Khan . 2015. “Replacement of Cow Milk *Chhana* With Soy *Chhana* in the Preparation of *Rasomalai* .” Bangladesh Journal of Animal Science 44: 59–63.

[fsn371145-bib-0011] Islam, M. S. , and S. Basak . 2013. “Study on Sweetmeat Processing in Bangladesh.” International Journal of Engineering & Science 2: 46–54.

[fsn371145-bib-0012] Islam, M. Z. , S. M. R. Rahman , M. M. Alam , and A. K. M. A. Mannan . 2003. “Manufacture of *Rassomalai* and Its Quality Attributes: An Indigenous Milk Sweetmeat of Bangladesh.” Pakistan Journal of Nutrition 2: 300–304.

[fsn371145-bib-0014] Jonas, J. J. , and J. F. Proctor . 2018. “Milk and Milk By‐Products.” In Handbook of Nutritional Supplements, 133–252. CRC Press.

[fsn371145-bib-0015] Jonkman, M. J. , and H. Das . 1993. “Optimization of Process Parameters for Production of *chhana* From Low Fat Cow Milk.” Journal of Food Science and Technology‐Mysore 30: 417–421.

[fsn371145-bib-0016] Joshi, S. V. , S. V. Majgaonkar , and V. A. Toro . 1991. “Effect of Different Coagulants on Yield and Sensory Quality of *Chhana* Prepared From Milk of Cow, Buffalo and Goat.” Indian Journal of Dairy Science 44: 380–383.

[fsn371145-bib-0017] Katiyar, P. N. , J. P. Singh , and P. C. Singh . 2008. “Effect of Zinc Sulphate, Borax and Calcium Nitrate in Flowering, Fruiting, Yield and Quality of Litchi cv.” Journal of Rural and Agricultural Research 8: 49–51.

[fsn371145-bib-0018] Khademi, F. , S. N. Raeisi , M. Younesi , et al. 2022. “Effect of Probiotic Bacteria on Physicochemical, Microbiological, Textural, Sensory Properties and Fatty Acid Profile of Sour Cream.” Food and Chemical Toxicology 166: 113244. 10.1016/j.fct.2022.113244. 35728727

[fsn371145-bib-0021] Mathur, G. K. , and V. B. Singh . 2001. “Protein Enriched *Rosogolla* .” Indian Journal of Dairy Science 54: 305–310.

[fsn371145-bib-0023] Mohamed, E. F. , and N. R. Attala . 2017. “Evaluating the Quality of *Rasogulla* as Cheese Balls in Sugar Syrup Prepared by Different Milk Types.” Egyptian Journal of Food Science 45: 11–16.

[fsn371145-bib-0024] Prodhan, U. K. , M. Alam , A. Sultana , et al. 2017. “Quality Assessment of Sweetmeat (*Rosogolla*) of Dhaka and Tangail Region of Bangladesh.” IOSR Journal of Environmental Science, Toxicology and Food Technology 11: 6–11. 10.9790/2402-1107010611.

[fsn371145-bib-0025] Qureshi, T. M. , G. Mueen‐ud‐Din , M. Nadeem , et al. 2024. “Effect of Different Preservatives on the Physicochemical Characteristics and Shelf Stability of *Rasmalai*: A Comparative Study.” Food Science & Nutrition 12: 3508–3515. 10.1002/fsn3.4019. 38726400 PMC11077211

[fsn371145-bib-0026] Qureshi, T. M. , M. Nadeem , G. Muhammad , et al. 2025. “Physico‐Chemical Characteristics, Antioxidant Activities, and ACE Inhibitory Potential of Pakistani *Dahi* Supplemented With Date Fruit Paste.” Food Science & Nutrition 13: e4588. 10.1002/fsn3.4588. 39898128 PMC11782915

[fsn371145-bib-0027] Sayedatunnesha, M. , A. Wadud , M. N. Islam , M. A. Islam , and M. A. Hossain . 2008. “Comparative Study of the Quality of *Rasomalai* Manufactured From Cow and Buffalo Milk.” Bangladesh Journal of Animal Science 37: 57–62.

[fsn371145-bib-0028] Sharma, S. P. , C. M. Kapoor , S. Bisnoi , M. Rani , G. Jairath , and S. Khanna . 2015. “Scale of Production, Compositional, Physico‐Chemical and Sensorial Attributes of Market Samples of *Rasmalai* Available in Hisar City of Haryana, India.” Asian Journal of Dairy and Food Research 34: 18–22. 10.5958/0976-0563.2015.00004.4.

[fsn371145-bib-0029] Sharma, S. P. , C. M. Kapoor , S. Khanna , M. Rani , S. Bishnoi , and S. S. Ahlawat . 2014. “Technological Aspects of Indigenous *Chhana* Based *Rasmalai* .” HaryanaVet 53: 124–126.

[fsn371145-bib-0034] Tarafdar, S. U. , M. A. H. Pramanik , B. Basak , M. S. Rahman , and S. K. Biswas . 2002. “A comparative study on the quality of *Rasogolla* made in laboratory and collected from local markets of Mymensingh, Bangladesh.” Pakistan Journal of Nutrition 1, no. 3: 156–160.

[fsn371145-bib-0030] Tyagi, S. , Y. Kumar , and D. Panwar . 2017. “Determination of Fat and Protein Contents of Khoa and *Chhana* Based Sweets.” International Journal of Applied Current Research 1: 1–10.

[fsn371145-bib-0031] Uzun, P. , F. Masucci , F. Serrapica , et al. 2018. “The Inclusion of Fresh Forage in the Lactating Buffalo Diet Affects Fatty Acid and Sensory Profile of Mozzarella Cheese.” Journal of Dairy Science 101: 6752–6761. 10.3168/jds.2018-14710. 29803420

[fsn371145-bib-0032] Wild, N. 2025. Everything You Need to Know About Color – Delta E. https://blog.globalgraphics.com/everything‐you‐need‐to‐know‐about‐color‐delta‐e/#:~:text=What%20is%20Delta%20E%20%E2%88%86,when%20printing%20textiles%20or%20posters.

[fsn371145-bib-0033] Zielińska, D. , B. Bilska , K. Marciniak‐Łukasiak , et al. 2020. “Consumer Understanding of the Date of Minimum Durability of Food in Association With Quality Evaluation of Food Products After Expiration.” International Journal of Environmental Research and Public Health 17, no. 5: 1632. 10.3390/ijerph17051632. 32138334 PMC7084339

